# Methylation of 45S Ribosomal DNA (rDNA) Is Associated with Cancer and Aging in Humans

**DOI:** 10.1155/2021/8818007

**Published:** 2021-01-28

**Authors:** Fengqing Shao, Xiaoqi Liu, Xianzhi Zhang, Qi Wang, Wencai Wang

**Affiliations:** ^1^Science and Technology Innovation Center, Guangzhou University of Chinese Medicine, Guangzhou 510405, China; ^2^Institute of Clinical Pharmacology, Guangzhou University of Chinese Medicine, Guangzhou 510405, China; ^3^Department of Horticulture, College of Horticulture and Landscape Architecture, Zhongkai University of Agriculture and Engineering, Guangzhou 510225, China

## Abstract

Cancer and aging, two distinct processes of cell development, are two major problems threatening our human health and life in current days. Epigenetic studies, especially DNA methylation, have been intensively investigated on them over the years, though a lot of unanswered issues remain. In the human genome, rDNA is a highly conserved tandem repeat family playing critical roles in protein synthesis, genome stability and integrity, etc. More importantly, rDNA is the significant target of DNA methylation, and a potential association between rDNA methylation and cancer and aging has emerged recently. However, whether there is a general trend that rDNA methylation is associated with cancer and aging remains an open issue. In this study, the involvement of rDNA methylation in a series of records of cancer and aging was investigated and summarized, upon which perspectives about rDNA methylation in cancer and aging were proposed. The results showed that rDNA methylation in most cancer cases displayed a consistent pattern with hypermethylation in the coding region but with hypomethylation in the promoter region, which likely facilitates the proliferation and metastasis of cancerous cells. Distinctively, both the coding and promoter regions of rDNA become increasingly methylated during the process of aging, indicating the decline of rDNA activity. The finding of rDNA methylation also implies its potential application as an epigenetic biomarker in the diagnosis of cancer and aging. This work will shed light on our understanding of the pathogenesis, diagnosis, and treatment of cancer and aging from the perspective of rDNA methylation.

## 1. Introduction

**Figure 1:**
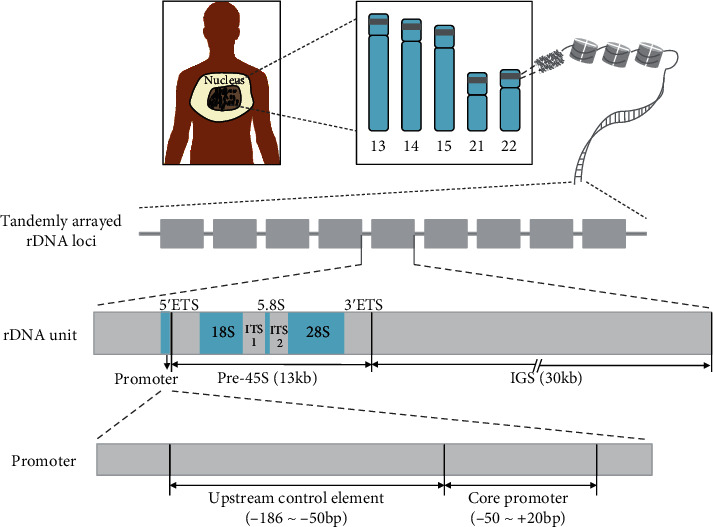
Structural organization of the rDNA clusters in the human genome showing the tandem arrangement of the multiple rDNA copies (above) and organization of each individual rDNA unit (middle) and the promoter region (bottom). ETS: external transcribed spacer; ITS1/2: internal transcription spacer 1/2; IGS: intergenic spacer. The promoter structure was redrawn from [15].

The highly conserved 45S ribosomal DNA (45S rDNA, hereafter referred to as rDNA for simplicity) is of importance for life since it plays critical roles not only in ribosome synthesis and global gene transcription and expression but also in the aspects of diseases, aging, genome stability, and evolution in the genomes of the vast majority of organisms [1–5]. In the human genome, rDNA are clusters of tandemly arrayed ribosomal RNA (rRNA) genes located at the short arms (p-arms) of five acrocentric chromosome pairs, i.e., chromosomes 13, 14, 15, 21, and 22 [6] (Figure 1). The mean copy numbers (CN) of rDNA in the human genome are c. 420 copies per diploid, although the intraindividual difference was found from c. 250 to 670 copies in a range of individuals [7]. Each human rDNA copy or unit (Figure 1) contains a 13 kb (kilobase) transcribed region that is transcribed into 45S precursor (45S-pre) rRNA and a 30 kb intergenic spacer (IGS) that is exclusive of transcription but contains substantial elements such as sequences of the promoter and terminator and subrepeats. rDNA sites on the chromosomes are claimed to be fragile sites for their highly repetitive structure and unstable feature, which could give rise to potential chromosome breaks, genetic diversity, and cellular senescence [6–9]. More importantly, it is noteworthy that rDNA sequences are significant targets of methylation with each rDNA unit containing c. >1500 CpGs (p represents phosphoric acid) or >10 CpGs per 100 nt in humans [10], which silences superfluous rDNA copies and regulates the activity of the nucleolar organizer region (NOR) where the rDNA copies are located [11–13]. Nevertheless, this is similar to other protein-coding genes, i.e., the inactive rDNA copies in general are hypermethylated, whereas the actively transcribed rDNA copies showed low or nonmethylation [14].

DNA methylation is a major and conserved epigenetic modification involving the addition of a methyl group (-CH_3_) to the 5^th^ carbon of the cytosine ring catalyzed by DNA methyltransferase (DNMT) converting cytosine into 5-methylcytosine (5mC). In the human genome, almost all the cytosine methylation occurs at the symmetric 5′-CpG-3′ dinucleotide context, which in most cases forms numerous CpG islands scattered across the whole genome. Additionally, direct DNA methylation can also occur on the N4 of cytosine and N6 of adenine, generating N4-methylcytosine (4mC) and N6-methyladenine (6mA), respectively [16]. But neither 4mC nor 6mA modification is frequently seen, e.g., 4mC was found in thermophilic bacteria, archaea, and mesophilic bacteria [17–20], whereas the function of 6mA that was found in both unicellular organisms and some multicellular organisms remains to be explored [21]. Nevertheless, 5mC is the best studied and most abundant DNA methylation type in a number of eukaryotes including us human beings; therefore, in this work, we lay our focus on the 5mC.

As the most common epigenetic modification, 5mC plays a critically important role in a range of aspects, e.g., chromosome structure maintenance, gene imprinting, tumorigenesis, and aging in the human genome [21]. Increasing studies demonstrated that there are links between the development or progression of human diseases and the DNA methylation pattern of certain genes [22–28]. In contrast, fewer studies have paid attention to the potential association between DNA methylation (5mC) of the rDNA sequences and diseases in human beings [15, 29–31], although people have realized that the integrative roles of rDNA are increasingly critical [32] and that rDNA are one of the key territories of methylation in the human genome [10]. Importantly, distinct from most other organisms whose large-sized rDNA unit has hardly been elucidated, the sequence and structure of the whole rDNA unit (Figure 1) in the human genome have already been successfully assembled, which provides advantages for further investigations on rDNA methylation in the human genome.

Various cancers are thought to be the major diseases causing deaths and are the most essential barrier to long life expectancy in the 21^st^ century [33]. It is reported that there were c. 14.1 million new cancer cases and 8.2 million deaths across the world in 2012 [34], which will expectedly grow into >20 million new cases by 2025 [35]. In addition to cancers, aging has become another huge threat to the health and longevity of human beings as well, for the naturally accumulated deleterious events such as genomic damage and for the occurrence of aging-related diseases, e.g., neurodegenerative diseases [36, 37]. Impressively, cancers are derived from a group of highly developed cells that grow vigorously and uncontrollably, while cells in the process of aging become senescent and far less dynamic, reflecting two distinct processes of cell development.

Collectively, considering the necessity to explore the association between rDNA methylation and cancer and aging in humans, here, we collected and summarized the rDNA methylation status in a range of human cancers and aging, based on which we then proposed perspectives to evoke more attention to the utility of rDNA in the diagnosis and even treatment of such diseases and to reveal the possible pathogenesis of diseases.

## 2. rDNA Methylation and Cancer

The occurrence of cancers was thought to be the result of gene deletion, mutation, and amplification that could lead to cell growth and differentiation barriers, whereas the precise pathogenesis of cancers remains unknown. Here, we collected several cases showing the association between the occurrence of cancers and rDNA methylation (Table 1).

### 2.1. rDNA Sequences: High Methylation

Chan et al. [38] investigated the methylation level of both 18S and 28S rDNA in 74 samples of late-stage ovarian cancer patients, nine samples of normal ovarian tissue adjacent to tumor sites, and 11 normal ovarian surface epithelial samples. They found that the hypermethylation status of both18S and 28S rDNA is highly correlated with ovarian tumors [38]. Moreover, they subsequently revealed that the rDNA methylation level was higher in ovarian cancer patients who have long progression-free survival than those with short survival [38]. These probably indicate that the hypermethylation status of rDNA sequences could serve as a potential diagnostic and prognostic marker in ovarian cancer.

To determine whether rDNA could be used as a potential indicator during breast tumor progression, Yan et al. [39] examined the methylation status of 18S, 5.8S, and 28S rDNA sequences in 58 primary breast cancer patients having partial or complete mastectomies and 10 healthy control individuals. They found that the overall methylation level of the above rDNA regions increased in c. 80% of the patients compared with the control samples, and the mean percentage of rDNA methylation was significantly higher (*P* < 0.0001) in patients with breast tumors than in the control group [39]. This is indicating that the rDNA hypermethylation status could be used as a potential marker in breast tumors. Yan et al. [39] also observed increased rDNA methylation in the primary stage, i.e., the poorly or moderately differentiated stage of breast cancer. However, this was contradictory to the results. Chan et al. [38] found that the rDNA methylation level was higher in ovarian cancer patients who have long progression-free survival than those with short survival. This reminds us that the course of the disease is critical and may be disease-specific when the rDNA methylation status is used as a potential marker in diagnosis, which undoubtedly should be taken into consideration.

Powell et al. [40] estimated the methylation level of the 5.8S and 28S rDNA using Southern blot in both the tumor and normal tissues of 215 female patients (30-93 years old) with primary endometrial carcinoma with the samples collected at the time of hysterectomy. It turned out that the rDNA methylation status in tumor patients was correlated with their therapeutic outcome: the majority of the patients (74%) had hypermethylated rDNA sequences and better disease-free survival and overall survival rates, but those who had a low level of rDNA methylation appeared to have significantly worse disease-free survival and overall survival rates. The authors also assessed other factors and their association with the rDNA methylation level that might influence tumor outcomes, such as racial differences and the use of adjuvant therapy, though multivariate analyses suggested that the rDNA methylation level was the only significant prognostic factor [40]. Nonetheless, this manifests that the methylation status of rDNA sequences may serve as a prognostic indicator for endometrial carcinoma patients on the premise of taking specific cancer stages into account, as previously stated [38, 39].

Impressively, Powell and colleagues [40] also found that there was a dramatically racial difference in the rDNA methylation levels between the African-American patients and the Caucasian patients, with the former more likely having low rDNA methylation levels. The disparity of methylation is interestingly similar to the findings. Wang et al. [4] discovered that in colorectal carcinoma (CRC), the African-American patients had c. 15-fold difference between the hypermethylated genomic regions (1588) and the hypomethylated region (100), whereas this difference among the Caucasian American CRC patients escalated to c. 25-fold [4]. The distinct racial disparity of rDNA methylation was also seen in plants [29] that displayed adaptive methylation patterns of rDNA across different geographical regions. This disparity might result from varied dietary restrictions or environmental factors that give rise to the different epigenetic landscape.

Moreover, Zhang et al. [30] analyzed the overall rDNA methylation levels in different cancers and normal tissues. The rDNA region was divided into five zones, i.e., Zone 1 (including the 5′ETS, transcribed regions, and 3′ETS) and Zones 2-5 (all belonging to the IGS region), without overlaps according to their CpG sites [37]. They found that the methylation levels of a majority of the CpG sites within Zone 2 (upstream of 5′IGS) and Zone 4 (downstream of 3′IGS) regions were significantly hypomethylated in four types of cancers including the liver, lung, prostate, and colon in comparison with the normal tissues. Besides, the variation trend of methylation levels in Zones 2 and 4 in the above four cancer types was overall consistent with that in the normal tissues, contrasting with the highly varied methylation levels in Zones 1, 3, and 5 [30]. The authors thus suggested that the methylation status of Zones 2 and 4, i.e., partial sequences of IGS, could serve as a useful marker for certain cancer detection in plasma [30].

### 2.2. rDNA Promoter: Low Methylation

In contrast to the hypermethylation of rDNA sequences in cancers, the promoter region of rDNA is usually hypomethylated in cancers. Zhou et al. [31] investigated the methylation status of the rDNA promoter region during the development of cervical cancer (cervical intraepithelial neoplasia (CIN)) in 10 patients. They found a much lower level of methylation of the rDNA promoter in the CIN tissues than in the normal tissues. In the meanwhile, significant elevation of rRNA transcription in most of the CIN specimens compared with the normal tissues was also observed, suggesting a positive correlation between the decrease of rDNA promoter methylation and the increase of rRNA transcription during the development of CIN [31]. Likewise, studies in human hepatocellular carcinomas revealed significant hypomethylation in the rDNA promoter of the cancerous tissues compared with that of the normal control samples, accompanied by increased rRNA synthesis [2].

In general, the occurrence of cancer is accompanied by the increase of rRNA transcription [41]; for example, rRNA transcription in lung cancers, primary prostate cancers, and C-MYC-driven cancers was elevated compared with rRNA transcription in the normal tissues [15, 42]. Investigation in colorectal cancer (CRC) showed that the synthesis of 45S-pre rRNA was significantly greater in the primary CRC tumor samples and cancer cell lines [43]. It is speculated that the occurrence of cancer drives the increase of rRNA transcription, given that the cancerous cells are highly metabolized and extremely proliferated. This inevitably induces huge demand for the biosynthesis of ribosomes and proteins, both of which are in need of huge loads of rRNA transcripts. Alternatively, the biosynthesis of ribosomes is a driving force for cancerous cells since they are more susceptible to ribosome synthesis disruption than normal cells [41, 44].

On the contrary, rRNA transcription elevation is not necessarily due to hypomethylation of the rDNA promoter. There were observations that found the occurrence of cancer with varied changes in the methylation status of the rDNA promoter region. For instance, Uemura et al. [15] found no methylation change of the rDNA promoter but found significant increases in rRNA transcription in prostate cancer. Ha et al. [45] found no significant difference in the methylation status of the rDNA promoter between the primary oral squamous cell carcinoma (OSCC) and the normal tissue samples. Interestingly, even hypermethylation of the rDNA promoter was observed in breast cancer cell lines [46]. This is explainable since there are a number of mechanisms modulating rRNA transcription, such as increase in the proportion of the structurally relaxed euchromatic rDNA copies [47], while a lowered methylated-rDNA promoter is merely one of them. Apart from direct effects to rRNA biosynthesis alternations attributed to rDNA promoter hypomethylation, there are indirect effects mediated by the rDNA promoter. For example, in gastric cancer, a tumor suppressor Zinc-finger protein 545 acts as the inhibiting factor of rRNA transcription via direct binding to the rDNA promoter region, inducing recruitment of other corepressors and histone modification changes, i.e., change from the transcriptionally active histone modification marker H3K4me3 relating to relaxed chromatin into the transcriptionally repressive histone modification marker methylated H3K9 associating with chromatin condensation and heterochromatin maintenance [48]. Moreover, the expression of a novel histone H4 variant H4G also altered chromatin conformation to enhance rRNA synthesis and subsequently cell growth in breast cancer [49].

Other plausible interpretations for the above inconsistent association between the rDNA promoter methylation status and cancers could be (1) varied progression of the disease stage of the examined samples as previously discussed and (2) varied sample types examined, i.e., clinical specimen tissues *in vivo* or cell lines *in vitro*. Nevertheless, we deduce that rDNA promoter hypomethylation triggers conformational changes of chromatin, i.e., increase chromatin accessibility and subsequent transcription elevation.

It is noteworthy that the hypomethylation of the rDNA promoter and the occurrence of cancer have no gender preference though some cases were from cervical cancer, breast cancer, and endometrial cancer that originally occurred in females.

## 3. rDNA Methylation and Aging

Aging is a time-dependent process accompanied by a series of physiological and metabolic function decline and a set of disease risk increase. It results from a combination of stochastic events such as genetic, epigenetic, and environmental influence, which give rise to changes in gene expression, chromosome stability, and so on.

As the cellular marker of longevity, NOR size shrank along with age, suggesting that aging might result from NOR loss and vice versa [50, 51]. The global 5mC DNA methylation level generally decreases during fruit development and ripening in plants, such as strawberry [52] and tomato [53], and during the natural aging processes in animals [54, 55]. But Huang et al. [56] unexpectedly found that global DNA methylation increases during fruit ripening in sweet orange. This indicates inconsistency of DNA methylation change during aging among different organisms. Herein, we focus on the potential association between aging and the status of rDNA methylation in the human genome, as rDNA methylation is emerging as an important aspect of the regulation of rRNA synthesis physiologically and pathologically, as mentioned above in cancer cases.

Numerous studies revealed a positive association between rDNA methylation and aging; that is, aging is accompanied by an escalation of rDNA methylation levels. It was evidenced that Swisshelm et al. [57] observed an age-related increase in the methylation of the 5′ end of 18S and 28S rDNA sequences in the murine brain, liver, and spleen in CBA/Ca mice beginning between 6 and 18 months. Oakes et al. [58] found that a region of the rDNA (5′-*NotI* site in the 5′ETS region) is preferentially hypermethylated with age in both spermatozoa and liver cells in rats. Also, the rDNA promoter region became hypermethylated during the process of replicative senescence [59]. Recently, Wang and Lemos [10] analyzed a set of published data generated from bisulfite sequencing of the whole blood tissue of mice at 16 different age stages from 0.67 to 35 months old to ask how the methylation level of each CpG site alters during the process of aging. They found that nearly 70% of the CpGs distributed at both the promoter and coding regions showed a positive correlation with age; in particular, the CpG site that was 10 nt downstream of the 5.8S rDNA was associated the most with age [10]. Moreover, their study suggested that the rDNA methylation was the most pronounced indicator among other genome components such as introns, exons, promoters, and repeats to reflect chronological age.

Differently, D'Aquila et al. [60] investigated the methylation status of the rDNA promoter in 472 human blood samples (20 to 105 years old) and in five different tissues of 15 rats (3–96 weeks old), finding no consistently significant correlation between the methylation of the rDNA promoter and chronological age in humans, but they indeed found an increased methylation level of the rDNA promoter in old rats [60]. However, the finding in human blood is debatable for the possible factors compromising DNA methylation monitoring, such as cellular heterogeneity of blood cells and subtype composition changes occurring physiologically and pathologically during aging [61, 62]. In addition, *in vitro* senescence of human fibroblasts from both normal donors and Werner syndrome patients was demonstrated to be associated with increased methylation of the 28S rDNA [63], indicating that methylation of rDNA might be a useful marker of aging both *in vivo* and *in vitro*. Collectively, the above findings indicate that methylation of rDNA can be applied as an optimal marker of aging in humans in contrast to other genomic components.

Impressively, when it comes to rDNA CN, Malinovskaya et al. [14] found that the CN of rDNA and methylation status changed during aging; that is, the normal elderly human group had their rDNA CN ranges narrowed (272 to 541 copies) in comparison with their young counterparts (200 to 711 copies). Moreover, almost no hypermethylated rDNA copies were detected in the examined elderly individuals, suggesting loss of hypermethylated rDNA copies during aging [14], which was also agreed by studying the cultured skin fibroblasts during their replicative senescence as well [14]. These results, from the view of evolution, suggest that the hypermethylated rDNAs, a class of zombie DNA in the genome, are probably prone to be selected during aging given that three categories of rDNA usually coexist in the human genome, i.e., nonmethylated (transcriptionally active), hypomethylated (transcriptionally inactive), and hypermethylated. And perhaps, it is according to this manner that the organisms are able to maintain their long life span.

## 4. Conclusion and Perspectives

**Figure 2:**
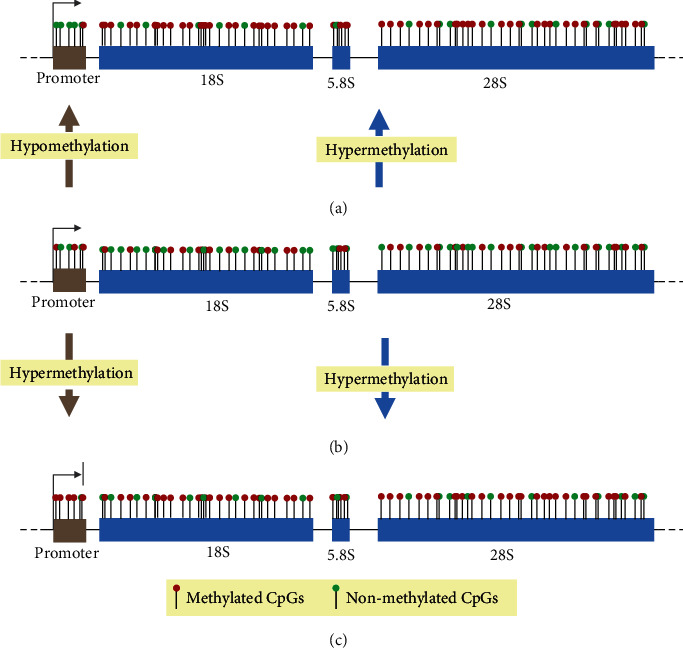
Schematic diagram of the rDNA methylation status between normal (b) and cancer (a) and aging (c) cells. Each vertical bar with circles in red and green represents methylated and non-methylated CpGs, respectively.

In this work, we reviewed and summarized the involvement of 5mC of rDNA in a series of research on cancer and aging. rDNA sequences in general maintain their normal status of methylation (Figure 2(b)) but in most cases display a consistent methylation pattern in cancers, that is, hypermethylation in the coding region but hypomethylation in the promoter region (Figure 2(a)), being in line with the gene body methylation paradox [64]. Distinctively, both the coding and promoter regions of rDNA become increasingly methylated during the process of aging (Figure 2(c)), suggesting a decline in rDNA activity.

It is not difficult to imagine that the varied methylation statuses of the rDNA promoter region in the two distinct cancerous and aging cells influence the synthesis of rRNA differently. That is, the low-methylated promoter in cancer promotes the synthesis of rRNA to meet their extensive demand, whereas the high-methylated promoter during aging inhibits the production of rRNA to cope with their physiological decline. However, it is speculated that the appearance of hypermethylation of rDNA sequences in cancer and aging cells is to improve and reduce the synthesis efficiency of rRNA, respectively. NORs are the busiest and most crowded territories within one genome executing transcription, DNA replication, repair, and so on, producing rRNA transcripts, the majority of cellular transcription within one living cell [25]. The dynamics of this process probably becomes even greater in the cancerous cells. Considering the large number of rDNA copies in each cell, up to tens of thousands, hypermethylation of selected rDNA copies can provide sufficient anchoring space for a series of transcription-related factors or change the chromatin configurations to speed up transcription and rRNA synthesis [53]. At the same time, increasing studies reveal a decrease in protein production, which is one of the main rRNA-consuming processes in the cell during aging [65]. We therefore inferred that in cancer cells, the efficiency of rRNA synthesis is greatly enhanced by the combination of the low-methylated promoter region and the high methylation of rDNA sequences, but in aging cells, rRNA synthesis is most likely inhibited by the high-methylated rDNA promoter and sequences themselves. In addition, medicines directed to rDNA methylation may be developed to provide epigenetic therapy as methylation inhibitors to treat cancer or slow down aging.

According to the above findings, we believe that there is a route of rDNA methylation that bridges upstream signal recognition and downstream regulations including chromatin alternation, rRNA synthesis, and genome stability, etc. to enhance or decline the potential deleterious influence caused by cancer or aging, respectively. The highly repetitive, heavily transcribed, and methylated features of rDNA make it a very fragile site in the genome, increasing the possibility of instability and further promoting the occurrences of cancer and aging and other relevant diseases [1, 66]. For instance, instability of rDNA was observed in cancers and premature aging syndromes of humans and yeast [66]. The extra-long IGS region, c. 30 kb in humans, within each rDNA unit is an ideal harbor accommodating fundamental elements that perhaps trigger changes of genome stability, cellular activities, or gene expression [67–70] that are involved in cancer and aging. In addition, sequence heterogeneity, CN alternation, or deamination of methylated rDNA may also be ascertained in cancer and aging, given that these aspects may also influence the stability of rDNA loci and even the whole genome [4, 5].

## Figures and Tables

**Table 1:** Summary of rDNA methylation in selected cancer cases.

Disease	rDNA region	Methylation status	References
Late-stage ovarian cancer	18S and 28S rDNA	Hypermethylation	Chan et al. [38]
Breast cancer	18S, 5.8S, and 28S rDNA	Hypermethylation	Yan et al. [39]
Primary endometrial carcinoma	5.8S and 28S rDNA	Hypermethylation	Powell et al. [40]
Liver cancer	IGS	Hypomethylation	Zhang et al. [30]
Lung cancer			
Prostate cancer			
Colon cancer			
Cervical cancer (cervical intraepithelial neoplasia (CIN))	Promoter	Hypomethylation	Zhou et al. [31]
Hepatocellular carcinomas	Promoter	Hypomethylation	Ghoshal et al. [2]
Primary oral squamous cell carcinoma (OSCC)	Promoter	No significant difference between cancer patients and control samples	Ha et al. [45]
Prostate cancer	Promoter	DNA methylation level remained unchanged	Uemura et al. [15]
